# Mendelian randomization analysis on the dissecting causal relationships between gut microbiota, circulating metabolites, and colorectal cancer: Insights from the latest evidence

**DOI:** 10.1097/MD.0000000000049390

**Published:** 2026-07-03

**Authors:** Mengshi Chen, Xiaoqian Dong, Hao Zhang, Weilong Zhong, Bangmao Wang

**Affiliations:** a Department of Gastroenterology, Shengli Clinical Medical College of Fujian Medical University, Fuzhou, Fujian, China; b Department of Gastroenterology, Fujian Provincial Hospital, Fuzhou, Fujian, China; c Department of Gastroenterology and Hepatology, Tianjin Medical University General Hospital, Tianjin Institute of Digestive Diseases, Tianjin Key Laboratory of Digestive Diseases, Tianjin, China; d School of Medicine, Nankai University, Tianjin, China.

**Keywords:** causality, circulating metabolites, colorectal cancer, gut microbiota, Mendelian randomization

## Abstract

The gut microbiota (GM) plays a direct role in colorectal cancer (CRC), but much of the epidemiological evidence linking the gut microbiome to CRC risk stems from observational studies. It remains unclear whether the observed microbial changes are causes or consequences of CRC development, and the role of metabolites as potential mediators is also uncertain. We conducted bidirectional Mendelian randomization (MR) using aggregated GWAS data on GM and circulating metabolites to explore causal relationships with CRC. Additionally, mediation analyses, 2-step MR, and multivariate MR were conducted to identify potential mediating factors of circulating metabolites in this relationship. We identified 12 positive and 15 negative causal effects between GM and CRC, and 4 positive and 3 negative causal effects between circulating metabolites and CRC. Notably, Succinivibrionaceae protected against CRC by increasing the CLA/FA ratio (CLA/FA; odds ratio [OR]: 1.045, 95% confidence interval [CI]: 1.006–1.086, *P *= .025), while *Peptococcus* increased CRC risk by raising the cholesterol esters to total lipids ratio in chylomicrons and extremely large VLDL (XXL-VLDL-CE_percent; OR: 1.098, 95% CI: 1.004–1.201, *P *= .04). This MR study provides new evidence supporting causal relationships between specific GM and CRC, along with potential new mediating metabolites.

## 1. Introduction

Colorectal cancer (CRC) ranks as the second most prevalent contributor to cancer-associated fatalities among women and the third among men, contributing to 10% of cancer mortality worldwide.^[1](p185)^ By 2035, it is expected that the incidence of CRC in developing nations will escalate to 2.5 million cases. The colorectum, abundant in microbiota, is the gastrointestinal tract most commonly affected by cancer in economically developed countries.^[2]^

Colorectal cancer development involves a complex interaction of environmental and genetic factors.^[3]^ Among the various microbial communities in the human body, the gut microbiome has received significant attention.^[4]^ Many investigations suggest that gut microbiota (GM) impacts intestinal and systemic health through metabolites, particularly short-chain fatty acids and lipopolysaccharides.^[5,6]^ A landmark 1967 study demonstrated that the carcinogenic effects of cycasin in conventional rats were influenced significantly by the presence of intestinal microorganisms, which did not induce cancer in germ-free rats.^[7]^ Subsequent research suggested that GM, encompassing species such as *Bacteroides*, *Clostridium*, *Enterococcus*, and *Escherichia*, could promote colorectal carcinogenesis by enhancing 1,2-dimethylhydrazine-induced aberrant crypt foci.^[8]^ Moreover, several investigations have depicted that the microbiota associated with CRC is different from that found in healthy individuals. CRC-associated microbiota exhibits a decreased abundance of potentially protective taxa (*Roseburia*), an elevated presence of procarcinogenic taxa (*Porphyromonas*, *Fusobacterium*, *Escherichia*, and *Bacteroides*), and greater species richness.^[9,10]^

However, observational studies, often cross-sectional or case-control, have limitations in establishing causality due to potential confounding, reverse causality, and bias. The exact relationship between GM, circulating metabolites, and CRC remains unclear. Hence, the aim of this research is to explain these correlations and discover potential metabolites for early detection and treatment of CRC.

Mendelian randomization (MR) is an effective analysis that utilizes genetic variants as instrumental variables (IVs) for mitigating reverse causation and controlling confounders. This method enables stronger causal inferences between clinical outcomes and exposures.^[11]^ The latest findings validate the use of human genetic data on gut microbial traits for clinical research, enabling MR to establish causal associations between CRC and the gut microbiome.^[12]^ Two-step mediation analyses and a bidirectional MR study were carried out utilizing summary statistics accessed at the latest genome-wide association studies (GWAS) on CRC, circulating metabolites, and the gut microbiome to explore their interrelationships.

## 2. Materials and methods

### 2.1. Data source

Characteristics of corresponding GWAS data sources are described in Table S1, Supplemental Digital Content 1. The GWAS summary statistics for 473 gut microbial taxa were sourced via the NHGRI-European Bioinformatics Institute GWAS Catalog (https://www.ebi.ac.uk/gwas/downloads/summary-statistics). This data retrieval process focused on examining the combined influence of diet and host genetics on the human GM and disease incidence within a large and homogeneous cohort (n* *= 5959) with matched shotgun fecal metagenomes and human genotypes.^[13]^ This cohort comprises participants from the FR02 study of the FINRISK study, encompassing women and men (aged 25–74) from 6 regions of Finland.^[14-16]^ 473 bacterial taxonomic units, spanning 235 species, 128 genera, 59 families, 21 orders, 17 classes, and 13 phyla, were associated with at least 1 genetic variant. This extensive dataset lays a comprehensive groundwork for detailed analysis.

The GWAS summary-level data for circulating metabolites were retrieved from a dataset of 1,36,016 participants across 33 cohorts.^[17]^ The large dataset includes 233 circulating metabolites, with 213 lipid and lipoprotein parameters or fatty acids, and 20 non-lipid traits such as amino acids, ketone bodies, glycolysis/gluconeogenesis, fluid balance, and inflammation-related metabolites. Meta-analysis revealed significant genome-wide associations for all 233 metabolic traits, demonstrating extensive pleiotropy and polygenicity.

The colorectal cancer GWAS summary statistics were derived via the FinnGen Consortium R9 release (https://r9.risteys.finngen.fi/), encompassing 287,137 controls and 6509 CRC cases. To ensure the robustness of outcomes, rigorous adjustments were applied throughout the analysis, accounting for principal components such as age, gender, and genotyping batch variations.

### 2.2. Instrumental variables selection

**Figure 1.**
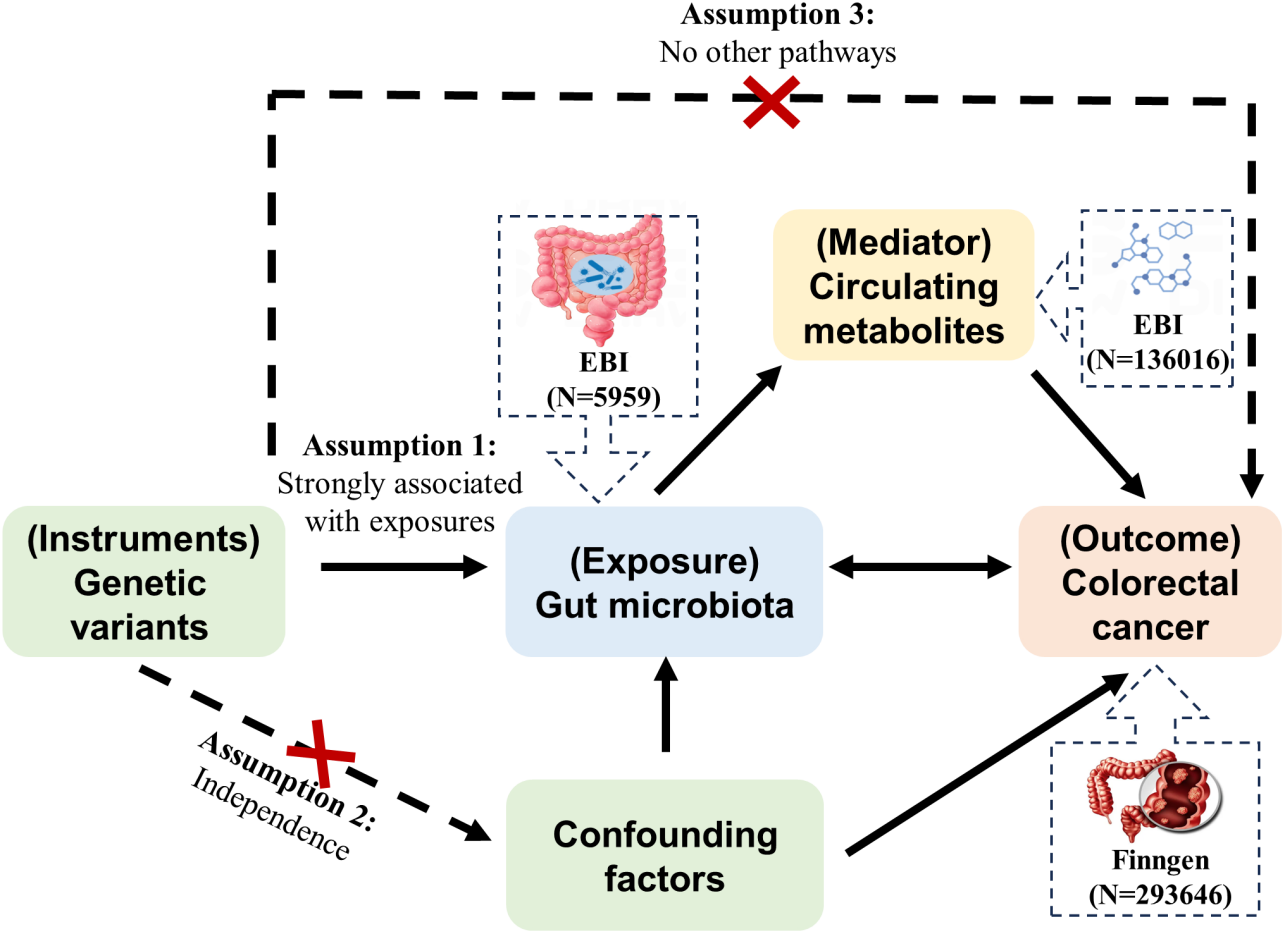
Hypothesis and design of bidirectional and mediated MR analyses. First, bidirectional UVMR was performed to investigate the causal relationship between gut microbiota and colorectal cancer. Second, 233 circulating metabolites (mediators) were selected for subsequent mediation analysis. Finally, a 2-step MR analysis was performed to detect potential mediating metabolites. EBI = European Bioinformatics Institute, MR = Mendelian randomization, UVMR = univariate Mendelian randomization.

In this research, a 2-sample MR analysis was conducted for validating the causal relationship. For ensuring the accuracy and robustness of the findings, quality checks were performed on the single nucleotide polymorphisms (SNPs) to acquire compliant IVs. The criteria for the selection of SNPs are mentioned below: the SNPs should demonstrate a strong association with exposures; the SNPs should not be linked to confounders; the SNPs should be related to exposure-mediated outcomes^[18]^ (Fig. 1).

For maintaining data reliability and ensuring a suitable number of SNPs for exposure analysis, a genome-wide threshold for gut microbial and circulating metabolites-related SNPs was set at 1 × 10^−5^. The standard GWAS thresholds (*P *< 5 × 10^−5^) were applied for CRC-related SNPs.^[19,20]^ Furthermore, a chained unbalanced aggregation method (window size > 10,000 kb, *r^2^* < 0.001) was utilized for eliminating unwanted SNPs, thereby maintaining their independence.^[21]^ SNPs exhibiting effect allele frequencies > 0.01 were selected, while those having *F*-statistics < 10 were removed to ensure data quality. The following formula was employed to calculate *F*-statistics.


$$F=\frac{R^{2}\left(N-k-1\right)}{\left[\left(1-R^{2}\right)k\right]}.$$

Herein, *R^2^* represents the fraction of variability demonstrated by every SNP. *N* denotes the GWAS sample size. Moreover, *k* represents the number of SNPs. An *F*-statistic of 10 demonstrates that the evidence regarding instrument bias is insufficient.^[22]^ The Benjamini–Hochberg method was used to correct the *P*-value and avoid false-positive rates in multiple hypothesis testing.^[23]^

### 2.3. MR analyses

#### 2.3.1. Two-sample univariate MR (UVMR) analysis

Two-sample MR analysis was employed for estimating the causal effect of GM and circulating metabolites on CRC (Fig. 1). Multiple analyses were employed, like MR Egger, inverse variance weighting (IVW), simple mode, weighted mode, and weighted median. The IVW analysis served as the significant approach, with the Wald ratios test applied to features consisting of a single IV.^[24]^ The MR findings were reported as odds ratios (ORs) along with their associated 95% confidence intervals (CI). The findings were considered statistically significant when the direction of MR-Egger and IVW were consistent and the *P*-value of IVW was < .05.

#### 2.3.2. Mediation MR analysis

For investigating the potential mediating role of circulating metabolites between CRC and GM, a 2-step MR (TSMR) method was employed.^[25]^ Initially, a UVMR was conducted for estimating the effect of GM on CRC (α). Following this, UVMR was utilized to detect circulating metabolites significantly correlated with CRC. The genetic impacts of GM were then adjusted via multivariate MR (MVMR) to examine the effect of these metabolites on CRC (β2). At last, UVMR was utilized for evaluating the impact of GM correlated with CRC on metabolites (β1). The calculation of the mediation ratio was carried out through β1*β2/α for quantifying the mediating effect of metabolites on the correlation between CRC and GM.

#### 2.3.3. Bi‑directional causality analysis

For the evaluation of the bi-directional causation effects between CRC and GM, CRC was treated as “exposure” and CRC-associated GM was utilized as “outcome.” The SNPs significantly related to dementia were selected (*P* < 5 × 10^−5^) as IVs.

### 2.4. Sensitivity analysis

MR-Egger regression was utilized to examine the possible impact of directed pleiotropy. *P* < .05 and nonzero regression intercept were considered statistically significant indicators of genetic pleiotropy. Furthermore, MR Pleiotropy RESidual Sum and Outlier (MR-PRESSO) analysis was carried out for detecting and correcting the effects from outliers.^[26]^ Cochran *Q* test was carried out to assess the heterogeneity of every SNP.^[27]^ Moreover, scatter plots of SNP–exposure and SNP–outcome associations were generated for visualizing MR outcomes.

### 2.5. Statistical analysis

R (v4.2.1; R Core Team) software was employed to perform all analyses. MR analysis was conducted utilizing the R packages “MendelianRandomisation” and “TwoSampleMR.” Additionally, “MR_PRESSO” was utilized for multiplicity tests (Ong and MacGregor, 2019).

### 2.6. Ethics approval

All GWAS summary statistics used in this study were obtained from publicly available databases that had received prior ethical approval.

## 3. Results

### 3.1. Instrumental variable selection

Initially, 30,661 SNPs linked to circulating metabolites and 9238 SNPs linked to GM, both reaching *P* < 1 × 10^−5^ (locus-wide significance level), were detected as potential IVs through large-scale GWAS following the removal of palindromic SNPs (Tables S2 and S3, Supplemental Digital Content 2). As mentioned in Table S4, Supplemental Digital Content 3, 127 SNPs were correlated with CRC in the FinnGen database. Significantly, all IVs displayed *F*-statistics > 10, indicating the absence of weak instrumental bias in the present research.

### 3.2. Bidirectional and 2-sample MR analyses of GM and CRC

**Figure 2.**
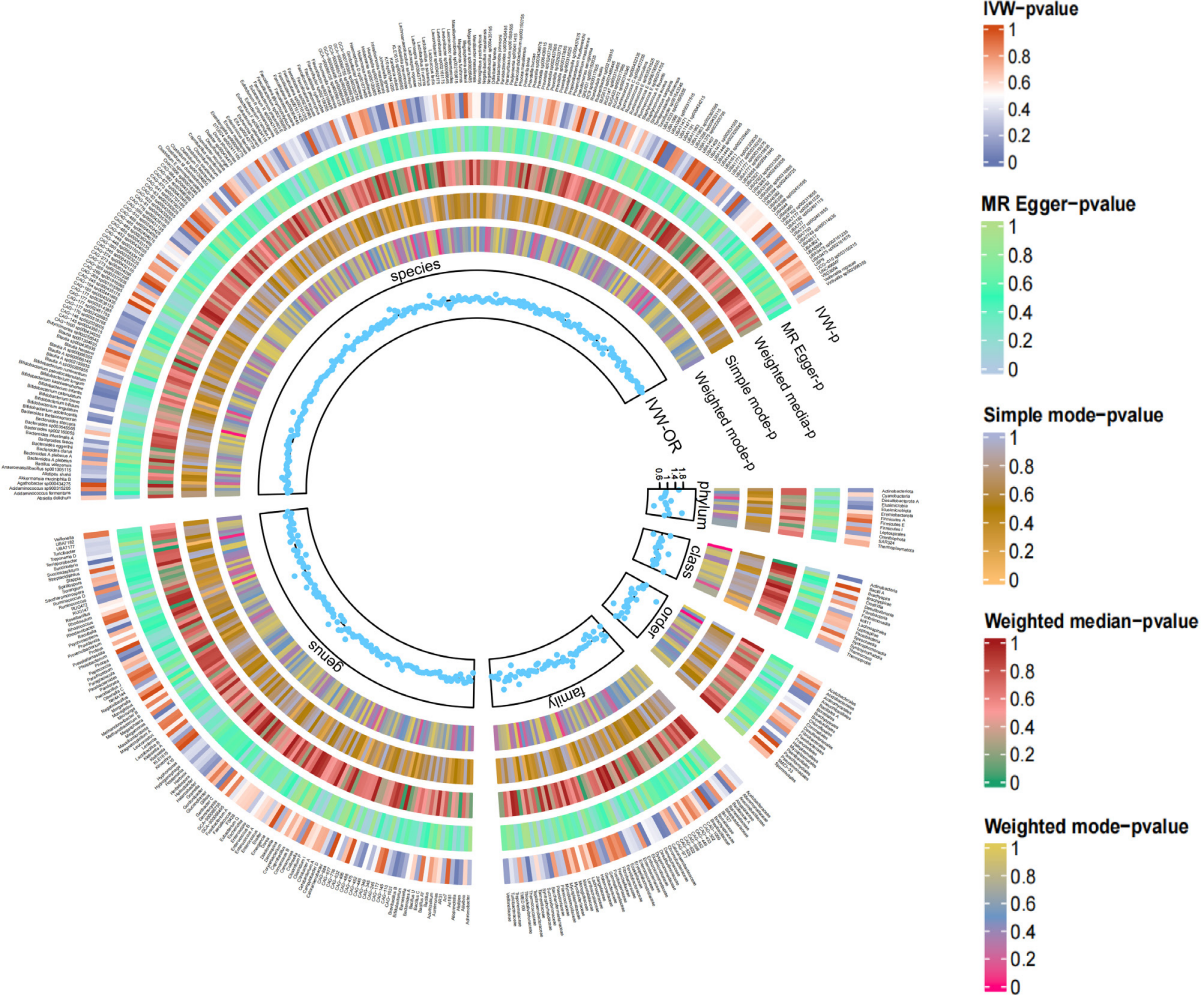
All results of MR analysis and sensitivity analysis between GM and CRC. CRC = colorectal cancer, GM = gut microbiota, IVW = inverse variance weighting, MR = Mendelian randomization, OR = odds ratio.

**Figure 3.**
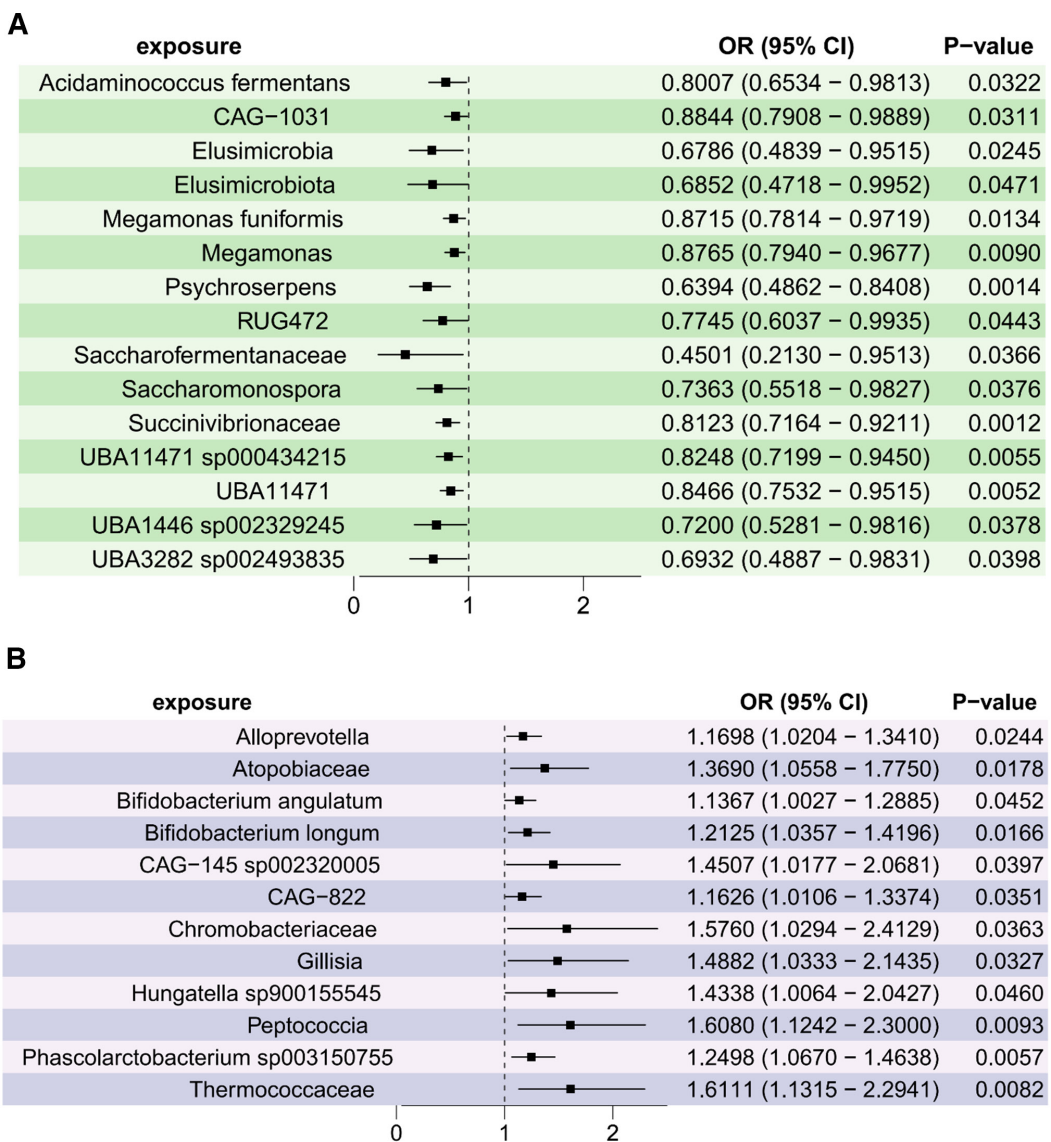
Forest plots summarizing the Mendelian randomization results of GM with a causal relationship to CRC using the IVW method (*P *< .05). (A) GM associated with reduced CRC risk. (B) GM associated with increased CRC risk. CI = confidence interval, CRC = colorectal cancer, GM = gut microbiota, IVW = inverse variance weighting, OR = odds ratio.

After conducting heterogeneity and pleiotropy tests utilizing Cochrane *Q* test and MR-Egger test, a total of 27 causal correlations were identified from GM characteristics (13 species, 6 genera, 6 family, and 2 phyla) to CRC traits via the IVW method (Figs. 2, 3 and Tables S5, Supplemental Digital Content 4). Notably, the genetically predicted *Acidaminococcus fermentans* (OR: 0.801, 95% CI: 0.653–0.981, *P* = .032), CAG-1031 (OR:0.884, 95% CI: 0.791–0.989, *P* = .031), Elusimicrobia (OR: 0.679, 95% CI: 0.484–0.951, *P* = .025), Elusimicrobiota (OR: 0.895, 95% CI :0.816–0.981, *P* = .047), *Megamonas funiformis* (OR: 0.871, 95% CI: 0.781–0.972, *P* = .013), *Megamonas* (OR: 0.877, 95% CI: 0.794–0.968, *P* = .009), *Psychroserpens* (OR: 0.639, 95% CI: 0.486–0.840, *P* = .001), RUG472 (OR: 0.774, 95% CI: 0.604–0.994, *P* = .044), Saccharofermentanaceae (OR: 0.450, 95% CI: 0.213–0.951, *P* = .037), *Saccharomonospora* (OR: 0.736, 95% CI: 0.552–0.983, *P* = .038), Succinivibrionaceae (OR: 0.812, 95% CI: 0.716–0.921, *P* = .001), UBA11471 sp000434215 (OR: 0.825, 95% CI: 0.720–0.945, *P* = .006), UBA11471 (OR: 0.847, 95% CI: 0.753–0.951, *P* = .005), UBA1446 sp002329245 (OR: 0.720, 95% CI: 0.528–0.982, *P* = .038), and UBA3282 sp002493835 (OR: 0.693, 95% CI: 0.489–0.983, *P* = .040) were linked to a decreased risk of CRC (Fig. 3A). Conversely, *Alloprevotella* (OR: 1.170, 95% CI: 1.020–1.341, *P* = .024), Atopobiaceae (OR: 1.369, 95% CI: 1.056–1.775, *P* = .018), *Bifidobacterium angulatum* (OR: 1.137, 95% CI: 1.003–1.288, *P* = .045), *Bifidobacterium longum* (OR: 1.213, 95% CI: 1.036–1.420, *P* = .017), CAG-145 sp002320005 (OR: 1.451, 95% CI: 1.018–1.068, *P* = .040), CAG-822 (OR: 1.163, 95% CI: 1.011–1.337, *P* = .035), Chromobacteriaceae (OR: 1.576, 95% CI: 1.029–2.413, *P* = .036), *Gillisia* (OR: 1.488, 95% CI: 1.033–2.144, *P* = .033), *Hungatella* sp900155545 (OR: 1.434, 95% CI: 1.006–2.043, *P* = .046), *Peptococcus* (OR: 1.608, 95% CI: 1.124–2.300, *P* = .009), *Phascolarctobacterium* sp003150755 (OR: 1.250, 95% CI: 1.067–1.464, *P* = .006), and Thermococcaceae (OR: 1.611, 95% CI: 1.131–2.294, *P* = .008) were related to a heightened CRC risk (Fig. 3B). After FDR adjustment of 473 gut microbiota, the results were no longer statistically significant (*P* > .05).

Following this, a reverse analysis was performed, revealing no evidence of a causal impact of CRC on the aforementioned GM (Table S6, Supplemental Digital Content 5).

### 3.3. Mediation analysis of potential circulating metabolites

**Figure 4.**
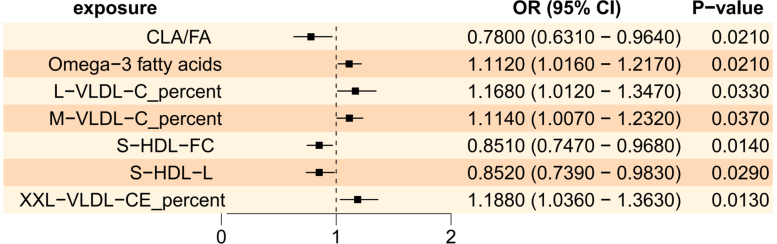
Forest plots summarizing the Mendelian randomization results of circulating metabolites with a causal relationship to CRC using the IVW method (*P *< .05). CI = confidence interval, CLA/FA = ratio of conjugated linoleic acid to total fatty acids, CRC = colorectal cancer, HDL = high-density lipoprotein, IVW = inverse variance weighting, L-VLDL-C_percent = total cholesterol to total lipids ratio in large VLDL, M-VLDL-C_percent = total cholesterol to total lipids ratio in medium VLDL, OR = odds ratio, S-HDL-FC = free cholesterol in small HDL, S-HDL-L = total lipids in small HDL, VLDL = very low-density lipoprotein, XXL-VLDL-CE_percent = cholesterol esters to total lipids ratio in chylomicrons and extremely large VLDL.

After excluding results with heterogeneity and pleiotropy, 7 circulating metabolites were identified as exhibiting a causal relationship with CRC (Fig. 4 and Table S7, Supplemental Digital Content 6). Specifically, free cholesterol in small HDL (S-HDL-FC; OR: 0.851, 95% CI: 0.747–0.968, *P* = .014), the ratio of conjugated linoleic acid (CLA) to total FAs (OR: 0.780, 95% CI: 0.931–0.964, *P* = .021), and total lipids in small HDL (S-HDL-L; OR: 0.852, 95% CI: 0.739–0.983, *P* = .029) were negatively associated with CRC. However, the cholesterol esters to total lipids ratio in chylomicrons and extremely large VLDL (XXL-VLDL-CE_percent; OR: 1.188, 95% CI: 1.036–1.363, *P* = .013), the total cholesterol to total lipids ratio in medium VLDL (M-VLDL-C_percent; OR: 1.114, 95% CI: 1.007–1.232, *P* = .037), the total cholesterol to total lipids ratio in large VLDL (L-VLDL-C_percent; OR: 1.168, 95% CI: 1.012–1.347, *P* = .033), and the levels of Omega-3 FAs (OR: 1.112, 95% CI: 1.016–1.217, *P* = .021) exhibited a positive correlation with CRC. The MR-Egger test depicted the absence of significant horizontal pleiotropy. After FDR adjustment of the 233 circulating metabolites, the results were no longer statistically significant (*P* > .05).

**Figure 5.**
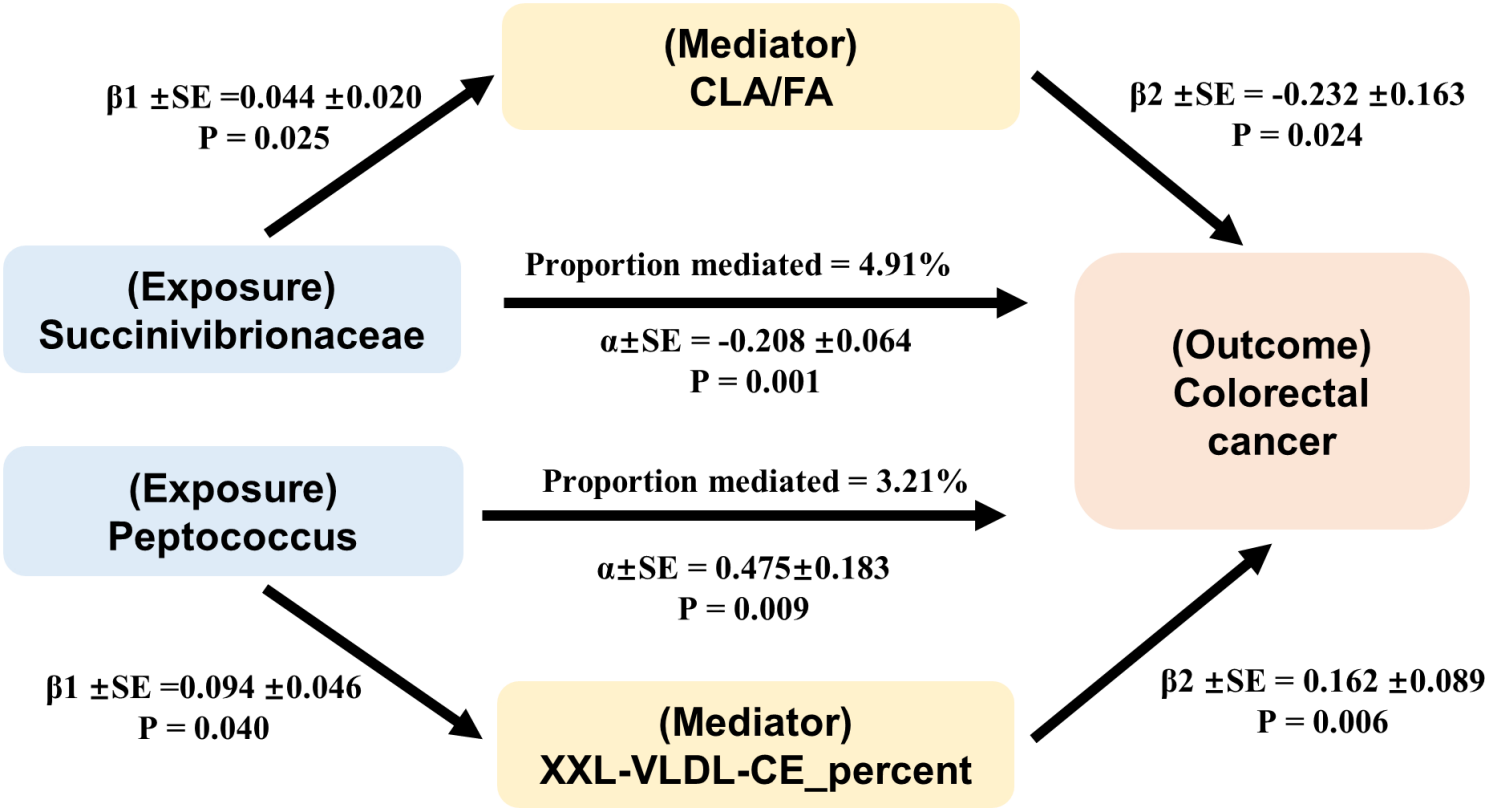
The figure shows the mediation pattern of “GM – circulating metabolites – CRC” in a TSMR and MVMR. β1 indicates the estimate of the causal effect of exposure on mediator via TSMR; β2 represents the controlled direct effects of each pair of bacteria and metabolite on CRC after adjusting for each other using MVMR; α is the total effect of exposure on CRC using the TSMR method. CLA/FA = ratio of conjugated linoleic acid to total fatty acids, CRC = colorectal cancer, GM = gut microbiota, MVMR = multivariate Mendelian randomization, SE = standard errors, TSMR = 2-step Mendelian randomization, VLDL = very low-density lipoprotein, XXL-VLDL-CE_percent = cholesterol esters to total lipids ratio in chylomicrons and extremely large VLDL.

Within the 27 GM causally linked to CRC, Succinivibrionaceae exerted protective effects against CRC by upregulating the ratio of CLA/FA (OR: 1.045, 95% CI: 1.006–1.086, *P* = .025). Conversely, *Peptococcus* exhibited detrimental effects on CRC by increasing XXL-VLDL-CE_percent (OR: 1.098, 95% CI: 1.004–1.201, *P* = .04; Table 1). The outcomes indicated no evidence of horizontal pleiotropy and heterogeneity. Furthermore, MVMR was employed to evaluate the independent effect of XXL-VLDL-CE_percent and CLA/FA on CRC. The calculation of the indirect effect and proportion influenced by these metabolites was carried out, revealing the significance of CLA/FA and XXL-VLDL-CE_percent after GM adjustment (Fig. 5). Overall, indirect effects of CLA/FA were observed in associations between Succinivibrionaceae and CRC, with a mediated proportion of 4.91%. Moreover, XXL-VLDL-CE_percent in the association between *Peptococcus* and CRC showed a mediated proportion of 3.21% (Fig. 5).

**Table 1:** Mendelian randomization analyses of the causal effects between gut microbiota and circulating metabolites.

Exposure	Outcome	Method	Nsnp	Beta ± SE	OR (95% CI)	*P*-value	*Q*-statistics	*P*-heterogeneity	Egger intercept	*P*-intercept
Succinivibrionaceae	CLA/FA	MR Egger	43	0.022 ± 0.042	1.023 (0.942–1.110)	.596	21.973	.993	0.003	.557
		IVW	43	0.044 ± 0.020	1.045 (1.006–1.086)	.025	22.323	.995		
Peptococcia	XXL-VLDL-CE_percent	MR Egger	17	−0.030 ± 0.075	0.970 (0.837–1.125)	.696	18.574	18.574	0.006	.067
		IVW	17	0.094 ± 0.046	1.098 (1.004–1.201)	.040	23.415	.103		

Beta, standard errors (SE), and *P*-values were obtained from the Mendelian randomization analysis. The heterogeneity test in the IVW method was performed using Cochran *Q* statistic.

CI = confidence intervals, CLA/FA = ratio of conjugated linoleic acid to total fatty acids, OR = odds ratio, IVW = inverse variance weighting, SE = standard error, VLDL = very low-density lipoprotein, XXL-VLDL-CE_percent = cholesterol esters to total lipids ratio in chylomicrons and extremely large VLDL.

## 4. Discussion

As per the assessed literature, this research represents the first implementation of MR utilizing a new dataset of 473 GM taxa and 233 circulating metabolites for exploring the causal relationship between GM and CRC, alongside investigating the potential mediating role of circulating metabolites. This investigation identified 27 unique taxa as well as a confirmed causal link to CRC. Additionally, TSMR was used to determine the intermediary function of circulating metabolites in the GM-CRC causal relationship. Significantly, *Peptococcus* was noted to exert a positive impact on CRC susceptibility, potentially through its mediation of elevated XXL-VLDL-CE_percent. Conversely, Succinivibrionaceae appeared to confer protection against CRC by increasing the CLA/FA ratio.

The intestinal microbiota, often regarded as a “forgotten organ,” is a complex and dynamic assembly of microbial communities residing in the human gut. These bacteria are crucial for maintaining digestive system homeostasis and host health, contributing to various immunological, protective, and metabolic functions.^[28]^ The phylogenetic composition and function of intestinal bacteria remain stable with age, whereas an increase in diversity is observed during growth. In healthy individuals, the large intestine hosts the most metabolically active and densest microorganisms, primarily consisting of bacteria from 4 main phyla: Actinobacteria, Bacteroidetes, Firmicutes, and Proteobacteria.^[29]^ In this study, Chromobacteriaceae and Succinivibrionaceae are categorized under Proteobacteria, while *Acidaminococcus*, *Hungatella*, *Megamonas*, *Peptococcus*, *Phascolarctobacterium*, and RUG472 belong to Firmicutes. Additionally, *Alloprevotella*, *Gillisia*, and *Psychroserpens* are classified under Bacteroidetes. Research indicates that the dominant phyla in the GM of patients having colorectal diseases are Proteobacteria, Firmicutes, and Bacteroidetes, which constitute over 95% of the microbial population in samples.^[30]^ However, variations in specific intestinal flora have been observed across different studies. An experimental study stratified CRC patients based on mucosal-associated bacterial co-abundance groups (CAGs), which resembled the concept of enterotypes. The study revealed an increase in Bacteroidetes Cluster 2, Firmicutes Cluster 2, Pathogen Cluster, and Allprevotella Cluster, whereas Firmicutes Cluster 1 and Bacteroidetes Cluster 1 exhibited a decrease in CRC mucosa.^[31]^ At the genus level, Alloprevotella was identified as a risk factor for CRC, aligning with previous research. The gram-positive bacillus *Bifidobacterium* participates in various physiological processes, including immunity, nutrition, digestion, and protection. Moreover, it is often regarded as a probiotic.^[32]^ However, our findings suggest that *B angulatum* and *B longum* promote CRC, contrary to other studies. Notably, the divergent associations observed for *B longum* may stem from strain-specific and host context effects. Whereas *B longum* subsp. infantis produces the anti-inflammatory metabolite indole-3-lactic acid,^[33]^ other subspecies and heat-inactivated preparations exhibit pro-apoptotic or immune-stimulatory activities in colon cancer models.^[34]^ Host genetic background, particularly APC mutation status, further shapes microbial impacts, as mutation burden correlates with Bifidobacterium abundances and epithelial Wnt signaling interactions.^[35]^ Similarly, although a high CLA/FA ratio has shown protective effects via PPAR-γ-mediated macrophage modulation in some settings,^[36]^ CLA supplementation has also been reported to accelerate azoxymethane/DSS-induced CRC through TGF-β- driven immunologic pathways.^[37]^ These complexities underscore the need for mechanistic follow-up studies that distinguish strain- and isomer-specific effects and that assess microbial–host interactions across diverse genetic and environmental contexts.

In this research, 15 GM components were revealed to potentially confer nominal protection against CRC. These encompassed *A fermentans*, CAG-1031, Elusimicrobia, Elusimicrobiota, *M funiformis*, *Megamonas*, *Psychroserpens*, RUG472, Saccharofermentanaceae, Saccharomonospora, Succinivibrionaceae, UBA11471 sp000434215, UBA11471, UBA1446 sp002329245, and UBA3282 sp002493835. Recent studies suggest that *Acidaminococcus* can predict immune-related adverse events (irAEs) and the effectiveness of immune checkpoint inhibitors in patients with CRC.^[38]^ Additionally, Elusimicrobia and Elusimicrobiota represent the same bacterium. Although direct evidence linking them to CRC is lacking, studies have shown reduced abundances of these phyla in gastric cancer,^[39]^ suggesting a protective role against gastrointestinal tumors, which is consistent with our findings. Research has depicted that *M funiformis* exerts a crucial function in distinguishing post-cholecystectomy patients from healthy controls and is involved in CRC progression.^[40]^ Succinivibrionaceae metabolizes to produce acetate and succinate, contributing to the synthesis of anti-inflammatory substances like vitamins. Succinate could enhance the production of interleukin-1β (IL-1β) by stabilizing hypoxia-inducible factor 1-alpha (HIF-1α), thus exhibiting anti-inflammatory effects.^[41]^ Additionally, succinate exerted antilipolytic action in adipose tissue by binding to SUCNR1, which inhibits the release of FAs from adipocytes.^[42]^ Furthermore, fat-stimulated bile acids and protein residues are metabolized by the microbiota into inflammatory and potentially carcinogenic metabolites, potentially enhancing the tumor progression risk.^[43]^ These mechanisms propose that Succinivibrionaceae may have a protective effect against CRC, which is consistent with our findings. Research exploring the relationship between CRC and the remaining bacteria is limited, highlighting the necessity for further investigation focusing on the in-depth exploration of these connections. This research exhibited a positive association between *Peptococcus* and CRC risk. Although direct investigations into the involvement of *Peptococcus* in CRC progression are lacking, its rise has been observed in patients with prostate and gastric cancers.^[44,45]^ Additionally, prior research indicated the association of a higher abundance of the genus *Peptococcus* with heightened levels of cholesterol and lipid in the plasma and liver,^[46]^ indicating that *Peptococcus* may contribute to CRC development. Multiple studies have reported the increased abundance of *Hungatella* in CRC, with high levels of *Hungatella* linked to a lower overall survival rate.^[47,48]^ Mechanistically, *Hungatella* has been implicated in reducing the sensitivity of CRC cells to 5-FU via downregulation of CDX2 expression.^[48]^ These outcomes present a basis for further exploration into the involvement and mechanisms of these bacteria in CRC.

The genetic evidence linking GM to 2 circulating metabolites has been provided through our mediation analyses. It was found that elevated levels of CLA/FA ratio are correlated with a reduced CRC risk. Succinivibrionaceae appears to influence CRC inhibition by increasing this ratio. CLA represents a collection of geometric and positional isomers of linoleic acid (LA; C18:2, c9, c12) featuring conjugated double bonds.^[49]^ CLA has shown diverse potential physiological characteristics, encompassing anticarcinogenic,^[50]^ anti-obesity,^[51]^ anti-cardiovascular,^[52]^ and antidiabetic activities,^[53]^ making it a promising food supplement.^[54]^ Additionally, many bacterial species, such as Propionibacterium acnes and Lactobacillus plantarum, are known for converting free LA into CLA.^[49]^ As per the assessed literature, no prior research has revealed a correlation between Succinivibrionaceae and CLA. Moreover, the pathways for CLA synthesis are not fully understood. Given that Succinivibrionaceae metabolizes to produce acetate and succinate, further exploration of its role in increasing the CLA/FA ratio is warranted. Additionally, these results indicate that *Peptococcus* potentially promotes CRC by increasing the cholesterol esters to total lipids ratio in extremely large VLDL and chylomicrons. Previous studies concerning the involvement of cholesterol esters in cancer have yielded mixed conclusions. For instance, some research suggests that elevated cholesterol levels may increase breast cancer risk, potentially through mechanisms involving oxidative stress and inflammation, which are linked to cancer progression.^[55]^ This aligns with our findings. Conversely, other studies suggest that cancer-derived cholesterol may prevent tumor infiltration by effector T cells, highlighting the complex nature of lipid metabolism.^[56]^ Cholesterol homeostasis is crucial for cellular and systemic functions, and disrupting this balance can trigger various pathological responses. The interaction between *Peptococcus* and lipid metabolism, as well as its role in tumor progression, requires further investigation. These findings, combined with our research outcomes, lay the groundwork for establishing a causal association between GM and the regulation of lipid metabolism.

It’s worth noting that CRC is a molecularly heterogeneous disease stratified into 4 consensus molecular subtypes (CMS1–4), each defined by distinct tumor microenvironment (TME) characteristics and microbial signatures.^[57,58]^ Specifically, CMS1 tumors are enriched in *Fusobacterium* species (e.g., *F nucleatum*, *F hwasookii*) and oral pathobionts such as *Porphyromonas gingivalis*, which promote myeloid cell infiltration and a pro-inflammatory microenvironment via FadA adhesin and LPS–TLR4 signaling.^[59]^ CMS2 is characterized by *Selenomonas* and *Prevotella* species correlating with integrin and Wnt/MYC pathway activation.^[60]^ CMS3 exhibits fewer strong microbial associations but involves taxa linked to lipid and carbohydrate metabolism, mirroring tumor metabolic rewiring.^[61]^ CMS4 displays stromal and EMT signatures alongside enrichment of *Porphyromonas asaccharolytica* and other oral-derived bacteria in inflamed tissues.^[62]^ Furthermore, emerging host–microbe interaction analyses reveal CMS-specific ferroptosis-related gene–microbe correlations, underscoring functional heterogeneity in CRC pathogenesis. These subtype-specific microbial features raise important interpretive considerations for our Mendelian randomization analysis, which necessarily treats CRC as a unified outcome due to limitations in publicly available GWAS summary data. Given the profound differences in microbial ecology and TME across CMS subtypes, the causal associations identified through MR may reflect subtype-specific effects averaged across heterogeneous tumor profiles. This highlights the need for cautious interpretation of microbiome–CRC associations and the future value of subtype-stratified GWAS and MR studies incorporating CMS annotations.

This study benefits from being the first to utilize the latest and most extensive GWAS summary data for circulating metabolites and GM. The sample size for circulating metabolites included more than 1,30,000 individuals across 33 cohorts, ensuring robust statistical power. Moreover, 2 methods (TSMR and MVMR) were employed for mediation analyses. Both methods supported the involvement of the cholesterol esters to total lipids ratio and CLA to FA ratio in the GM to CRC pathway, offering valuable insights into future clinical applications.

However, this research has certain limitations. Initially, the CRC patients and GM data assessed were exclusively from the Fenland study, thus restricting the generalizability of our findings to a broader population. Multiple studies demonstrate that core gut microbial profiles – including the *Prevotella*/*Bacteroides* axis, Succinivibrionaceae, *Peptococcus*, *Faecalibacterium*, and *Ruminococcus* – vary substantially across global populations in response to dietary habits, cultural practices, and environmental exposures.^[63-65]^ For instance, Succinivibrionaceae is enriched in Western high-fat diets, whereas *Peptococcus* predominates in high-fiber Asian cohorts^[64,65]^; rural versus urban lifestyles further modulate archaeal taxa such as *Methanobrevibacter smithii*.^[66,67]^ Such variability suggests that the magnitude or even direction of protective associations with CRC risk may differ outside European populations. Future MR analyses leveraging GWAS and metagenomic data from East Asian, African, Latin American, and other ancestries will be essential to validate and extend our findings across diverse dietary and microbial baselines. Additionally, given the exploratory nature of our study and our goal of uncovering additional taxa potentially causal for CRC, we therefore increased instrument count and statistical power by adopting a more permissive threshold of *P* < 1 × 10^−5^, a strategy frequently used in microbiome MR research.^[68-73]^However, this may introduce weak instrument bias, particularly for low-abundance taxa with limited associated variants. Although all retained SNPs exhibited *F* statistics above 10, satisfying conventional strength criteria, instruments comprising only 1 to 3 variants risk attenuation of causal estimates toward confounded observational associations. Future MR analyses should, where sample size allows, prioritize genome-wide significant instruments (*P* < 5 × 10^−8^) for common taxa and replicate findings across independent, ancestrally diverse cohorts to reinforce causal inference. Third, most associations did not remain significant after multiple testing correction. Given the large number of exposures, we proceeded with downstream analyses to preserve potentially meaningful signals. However, this approach may have increased the risk of false positives. Moving forward, validation in larger independent cohorts and the application of more rigorous multiple testing corrections will be essential to confirm these preliminary findings. Lastly, while we applied FDR correction to control for multiple metabolite testing, we note that our mediation proportions (4.91% and 3.21%) are modest and warrant careful interpretation. Small mediation fractions can reflect limited total effect sizes, measurement error, or residual confounding, as highlighted in simulation studies showing reduced reliability of mediation when total effects are small.^[74]^ However, in complex host–microbiome networks, even minor indirect effects may reveal key mechanistic nodes, particularly when corroborated by independent experimental or cohort data.^[75]^ Moreover, microbial–metabolite interactions frequently exhibit threshold behaviors that linear models cannot fully capture; future analyses incorporating nonlinear mediation frameworks with exposure–mediator interactions or spline functions will be crucial for uncovering true indirect effects in CRC pathogenesis.^[76]^

## 5. Conclusion

This study has identified causal relationships between GM, circulating metabolites, and CRC, proposing new mechanisms of GM action. The beneficial or detrimental GM identified may offer significant understanding into microbiota-mediated CRC pathogenesis, establishing effective measures for its prevention and management. This study presents novel strategies for metabolite- and microbiome-driven treatments and interventions in CRC.

## Acknowledgments

We appreciate the investigators of the original studies for sharing the GWAS summary statistics. We also acknowledge the assistance from medical writers, proof-readers and editors.

## Author contributions

**Conceptualization:** Mengshi Chen, Xiaoqian Dong, Weilong Zhong.

**Funding acquisition:** Weilong Zhong, Bangmao Wang.

**Methodology:** Mengshi Chen, Hao Zhang.

**Software:** Xiaoqian Dong.

**Writing – original draft:** Mengshi Chen, Xiaoqian Dong, Hao Zhang.

**Writing – review & editing:** Weilong Zhong, Bangmao Wang.














